# Identification and Characterization of Beneficial Soil Microbial Strains for the Formulation of Biofertilizers Based on Native Plant Growth-Promoting Microorganisms Isolated from Northern Mexico

**DOI:** 10.3390/plants12183262

**Published:** 2023-09-13

**Authors:** Carlos Esteban Guardiola-Márquez, María Teresa Santos-Ramírez, Melina Lizeth Figueroa-Montes, Eric Oswaldo Valencia-de los Cobos, Iván Jesús Stamatis-Félix, Diego E. Navarro-López, Daniel A. Jacobo-Velázquez

**Affiliations:** 1 Tecnologico de Monterrey, Escuela de Ingeniería y Ciencias, Ave. General Ramon Corona 2514, Zapopan 45138, Jalisco, Mexico; a01560073@tec.mx (C.E.G.-M.);; 2 Tecnologico de Monterrey, Institute for Obesity Research, Av. General Ramon Corona 2514, Zapopan 45201, Jalisco, Mexico

**Keywords:** bacteria, beneficial microorganisms, biofertilizers, fungi, native microbial strains, plant growth-promoting microorganisms, rhizosphere, rhizobacteria, symbiosis

## Abstract

Plant growth-promoting microorganisms (PGPM) benefit plant health by enhancing plant nutrient-use efficiency and protecting plants against biotic and abiotic stresses. This study aimed to isolate and characterize autochthonous PGPM from important agri-food crops and nonagricultural plants to formulate biofertilizers. Native microorganisms were isolated and evaluated for PGP traits (K, P, and Zn solubilization, N_2_-fixation, NH_3_-, IAA and siderophore production, and antifungal activity against *Fusarium oxysporum*). Isolates were tested on radish and broccoli seedlings, evaluating 19 individual isolates and 12 microbial consortia. Potential bacteria were identified through DNA sequencing. In total, 798 bacteria and 209 fungi were isolated. Isolates showed higher mineral solubilization activity than other mechanisms; 399 bacteria and 156 fungi presented mineral solubilization. Bacteria were relevant for nitrogen fixation, siderophore, IAA (29–176 mg/L), and ammonia production, while fungi for *Fusarium* growth inhibition (40–69%). Twenty-four bacteria and eighteen fungi were selected for their PGP traits. Bacteria had significantly (ANOVA, *p* < 0.05) better effects on plants than fungi; treatments improved plant height (23.06–51.32%), leaf diameter (25.43–82.91%), and fresh weight (54.18–85.45%) in both crops. Most potential species belonged to *Pseudomonas, Pantoea, Serratia,* and *Rahnella* genera. This work validated a high-throughput approach to screening hundreds of rhizospheric microorganisms with PGP potential isolated from rhizospheric samples.

## 1. Introduction

The plant rhizosphere is the soil area in close association with the plant root system; it provides a microenvironment for the plants to establish different types of interactions with soil microorganisms, which, depending on their characteristics, will positively or negatively affect the plant’s growth and development [1,2,3,4]. Rhizospheres can harbor a wide variety of microbial taxa, of which plant growth-promoting microorganisms (PGPM) represent a vital constituent of the soil ecosystems with fundamental biological and ecological functions [1,2,4,5]. Soil-beneficial microorganisms establish symbiotic relationships with plants; they colonize plant roots and benefit plant health by enhancing plant nutrient acquisition and assimilation and plant protection against various biotic and abiotic stresses [1,3,5,6,7,8]. In exchange, plants produce sugars, proteins, amino acids, flavonoids, fatty acids, and organic acids, release them through root exudates, and serve as energy or nutritional sources for microorganisms to grow and develop [1,3,9,10].

PGPM in the rhizospheric area greatly benefits plants by promoting plant growth through various direct and indirect mechanisms. Direct effects include enhancing plant nutrition by providing nutrients through atmospheric nitrogen (N_2_) fixation, ammonia (NH_3_) production, and solubilization of soil minerals (including phosphorus (P), potassium (K), zinc (Zn), and iron (Fe)), and improving plant growth by modulating phytohormone levels (including auxins, cytokinins, gibberellins, ethylene, and abscisic acid). Indirect modes of action involve improving plant health by suppressing phytopathogens via parasitism, nutrient and niche competition in the rhizosphere, production of antagonistic compounds (such as siderophores and antibiotics) and lytic enzymes (including proteases, glucanases, and chitinases), and stimulating plants systemic resistance against several phytopathogenic microorganisms [1,4,5,6,7,9,11].

Unfortunately, one of the main problems affecting the microbial equilibrium in the soil is modern intensive agricultural production practices, such as indiscriminate, uncontrolled, and excessive use of chemical fertilizers to maintain and increase agricultural productivity due to the growing pressure on food production generates, in addition to soil and groundwater pollution, a rupture in the interactions between plants and beneficial soil microorganisms [5,6,7,9,12]. Long-term use of fertilizers reduces soil microbial diversity. For this reason, it is important to re-enrich the rhizosphere with PGPM, which can restore the balance and symbiosis that allows the proper development of the plants [5,7].

Beneficial soil microorganisms can be isolated, characterized, identified, and propagated to be used in agriculture as biofertilizers. It is important to exploit the potential of native microorganisms, as several authors indicate that the efficiency of biofertilizers is higher when native strains isolated from the rhizosphere are used [11,12,13]. Successful biofertilization in the field is achieved with high colonization rates. It has been reported that indigenous microbial strains are more efficient in colonizing plant roots than laboratory or commercial strains; since root colonization and plant–microbial interactions are induced by the root and seed exudates, native microbial strains could be metabolically and genetically adapted to develop those processes [11,13,14].

There are different opinions regarding the optimal procedure to recover and select PGPM to develop commercial biofertilizers [4,6,14]. Vasseur-Coronado et al. [6] described and validated a strategy to select the best plant growth-promoting microorganisms. To start searching for effective strains for inocula preparation, it is required to determine the target crop and the commercial strategy. The next step consists of the isolation of soil microorganisms from different soil and root samples; in this step, the selection of growth media is very important since, depending on the growth media (i.e., differential, selective, nutrient-rich, or poor media), different microbial groups will be isolated; microbial candidates are commonly grown in enrichment culture media to recover a high number of microbial strains. Microorganisms are subsequently screened for multiple plant growth-promoting mechanisms using standard methods [4]. The following assays involve the evaluation of traits associated with successful product development, such as mass production on cheap growth medium, taxonomic identification, plant pathogenicity, efficacy testing in a bioassay, toxicological studies, and the assessment of their effects on plant growth in controlled seedbed tests to prescreen candidates without time-consuming or expensive methodologies, estimating their capacity to colonize the rhizosphere and plant roots, and to exhibit the beneficial traits. Final steps involve the evaluation of the plant growth-promoting efficacy of the best candidates under greenhouse and/or field experiments [6]. 

Nowadays, the distribution and use of native PGP microbial preparations in agriculture have significantly risen worldwide due to the beneficial effects on nutrient uptake, plant and soil health and crop productivity, the increasing cost of synthetic fertilizers, and the demand for eco-friendly technologies in the society [1,4,5,6]. Promoting agriculture sustainability through an increase in the utilization of efficient nutrient mobilizing PGPM is an important strategy to achieve high crop yields, better soil health, and lower chemical fertilization and environmental degradation [1,5,7]. Therefore, this research aims to isolate and characterize autochthonous microbial strains from important agri-food crops and nonagricultural plant species of northern Mexico with potential growth-promoting traits for the formulation of biofertilizers.

## 2. Results

### 2.1. Isolation of Native Soil Microorganisms and Evaluation of Plant Growth-Promoting Traits

The thirty collected samples resulted in ten composite soil samples and ten composite root samples, corresponding to the five plant species from two fields. Replicates were homogenized and mixed. Composite soil and root samples were inoculated on solid enrichment growth media (TSA for bacteria and PDA for fungi) to recover rhizospheric and endophyte microorganisms. In total, 798 bacterial and 209 fungal isolates were identified and subcultured based on visual morphological differences. A higher number of bacterial isolates were obtained because it was more challenging to recognize differences in pigmentation, texture, shape, size, opacity, and elevation of bacterial colonies than fungal isolates.

**Figure 1:**
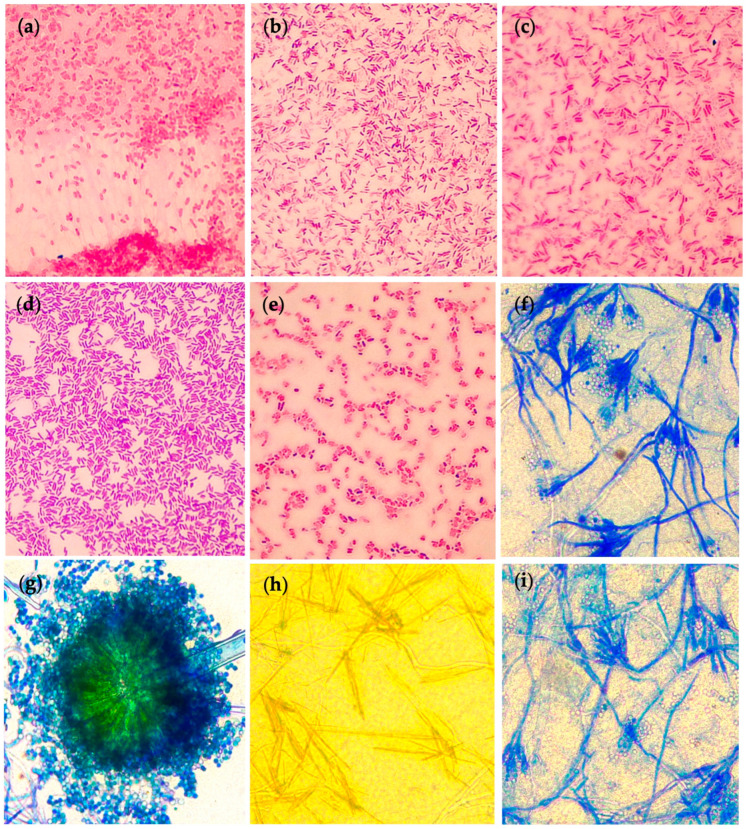
Microscopic images of bacterial (Gram-stained) and fungal (methylene blue-stained) isolates. (**a**) 58B bacterial isolate. (**b**) 196B bacterial isolate. (**c**) A316-B bacterial isolate. (**d**) B9M bacterial isolate. (**e**) A313–2 bacterial isolate. (**f**) H202_2 fungal isolate. (**g**) H97 fungal isolate. (**h**) H38 fungal isolate. (**i**) H56 fungal isolate. Magnification 100×.

Isolates were subjected to several selection rounds to select the best PGPM as potential active ingredients of biofertilizers. For this purpose, different PGP attributes were evaluated including potassium, phosphate, and zinc solubilization, nitrogen fixation, ammonia production, indole-3-acetic acid (IAA) secretion, siderophore production, and antifungal activity against phytopathogenic *Fusarium oxysporum*. For bacteria, from the 798 isolates, 50% (399 isolates) were discarded because they did not show P, K, and Zn solubilizing activity. Of the remaining 50%, 65% were selected because they showed at least two mineral (P, K, Zn) solubilizing parameters in level 3 (clearing zones ≥ 5 mm) and nitrogen-fixing activity. For the next step, NH_3_ production, IAA secretion, and siderophores production were included in the evaluation. Consequently, only 17% of the total initial bacteria were chosen. Selected isolates were screened for antifungal activity, and a last selection was made where only 24 bacteria (3% of total bacteria) were selected as final isolates for further analysis (Table 1). Isolates were Gram-stained, and results showed a predominance of Gram-negative bacteria (pink-red stained with safranin) and presented rod-shaped bacilli morphology (Figure 1). 

Regarding fungal selection, 209 fungal isolates were recovered, of which 75% (156 isolates) exhibited P, K, and Zn solubilizing activity. After screening for N_2_-fixation, IAA, NH_3_, and siderophores production, only 55 fungi continued in the characterization process. The following selection round was based on a second grouping of isolates depending on the macroscopic morphological similarity of the fungi, resulting in 34 fungal isolates with morphological differences (16% of the total initial isolates). Finally, eighteen isolates (9% of total fungi) were selected as the best PGP fungi for further analysis based on their multiple PGP mechanisms and biocontrol activity (Table 1). Strains were methylene blue-stained, observing that the most common morphologic characteristics corresponded to the *Penicillium* and *Aspergillus* genera: filamentous fungi with branched or simple conidiophores finishing in phialides arranged in a brush-like bunch [15], and conidiophores ending in a typical conidial head composed of one-celled circular conidia [16], respectively (Figure 1). In fungi, there was one less round of selection due to the number of isolates.

**Figure 2:**
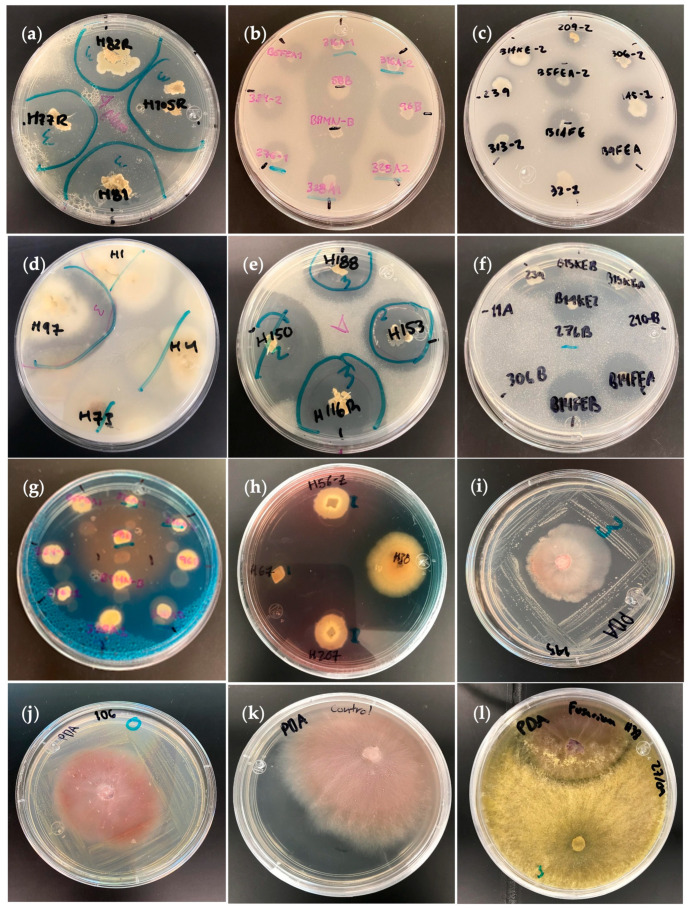
Plant growth-promoting traits evaluation. (**a**) Fungal isolates presenting potassium solubilization at level 3 (clearing halo ≥ 5 mm) in Aleksandrov medium. (**b**) Bacterial isolates with clear halo zones at level 3 (≥5 mm; isolates 58B, B9MN-B, 96B) and level 2 (2–4 mm; isolates 334-2, B5FEA1, 328 A1) for potassium solubilization. (**c**) Phosphate solubilization by bacteria in Pikovskaya (PVK) agar medium. (**d**) Positive and negative phosphate solubilization activity for fungal isolates. (**e**) Fungal zinc solubilization halos level 3 (≥5 mm) in modified PVK agar medium containing insoluble zinc oxide. (**f**) Zinc-solubilizing bacteria (level 2 and 3). (**g**,**h**) Orange-yellowish halos indicating siderophores production for bacterial and fungal isolates, respectively. (**i**) Bacterial strain A145 limiting the growth of the phytopathogenic *F. oxysporum*. (**j**) Bacterial isolate without biocontrol activity. (**k**) Isolated *F. oxysporum* as negative control. (**l**) The best fungus with biocontrol activity against *F. oxysporum*.

Microbial strains were classified into three levels for each PGP trait (Table 2). In general, microbial isolates showed higher mineral solubilization activity than the other PGP mechanisms. In this study, 399 bacterial strains were evaluated for mineral solubilizing activity, of which 11%, 25%, and 33% of bacterial isolates presented level 3 (clearing zone ≥ 5 mm) for P, K, and Zn solubilizing activity, respectively, while for fungi, from the 156 strains evaluated, 6%, 20%, and 46% exhibited level 3 (clearing halo ≥ 5 mm) for P, K, and Zn solubilization, respectively. Figure 2 shows the formation of halo zones in different sizes due to mineral solubilization (Figure 2a–f), siderophores production (Figure 2g,h), and antifungal activity (Figure 2i–l). 

Table 3 summarizes the results of the characterization of the final 24 bacterial and 18 fungal isolates selected for their plant growth-promoting traits. This summary clarifies that mineral solubilization activities predominated in both types of microorganisms, with fungi showing the largest solubilization halos, reaching 20 mm in the halo radius. Siderophore production was also higher in fungi, but a greater number of bacterial isolates presented levels between 2 and 3 (around 67% of the isolates). Regarding nitrogen fixation, ammonia production, and IAA secretion, bacteria stood out from fungi, which showed negligible results; bacteria produced the phytohormone IAA in ranges between 29 and 176 mg/L, while fungi did not exceed 10 mg/L. Finally, antifungal activity against phytopathogenic *Fusarium oxysporum* was more evident among the fungal isolates since around 66% of the fungi showed levels 2 and 3, with *Fusarium* growth inhibition ranging between 40 and 69%.

### 2.2. Preparation of Bio-Formulations and In Vivo Evaluation in a Seedbed Assay

Twenty-four selected bacterial isolates were individually grown in an enrichment medium. Plate count indicated that cell density was at 1.11–8.75 × 10^8^ CFU mL^−1^. Similarly, eighteen fungal isolates were propagated in the PDA agar medium, and spores were recovered in liquid suspensions with a final concentration of 1.80–8.9 × 10^6^ spores mL^−1^. Isolates were used to formulate six consortia for each type of microorganism, grouping those with greater relevance for each PGP attribute (Table 4). It is important to clarify that due to the selection process, all the strains have more than one mechanism at level 3; therefore, the isolates were distributed in such a way that there is an arrangement of microorganisms with a high level of activity for each PGP trait.

In total, twelve consortia (six bacteria and six fungal consortia) and nineteen individual microorganisms (fifteen bacteria and four fungi) were evaluated in a seedbed experiment for 12 days to analyze the early plant response of radish (*Raphanus sativus*) and broccoli (*Brassica oleracea*) to the bioformulations. Considering three replicates, data from nine plants per treatment were collected by measuring the parameters of plant height (cm), leaf diameter (cm), root length (cm), and fresh weight (g) of the sprouts.

#### 2.2.1. Early Plant Response of Broccoli Sprouts to Bio-Formulations in a Seedbed Assay

**Figure 3:**
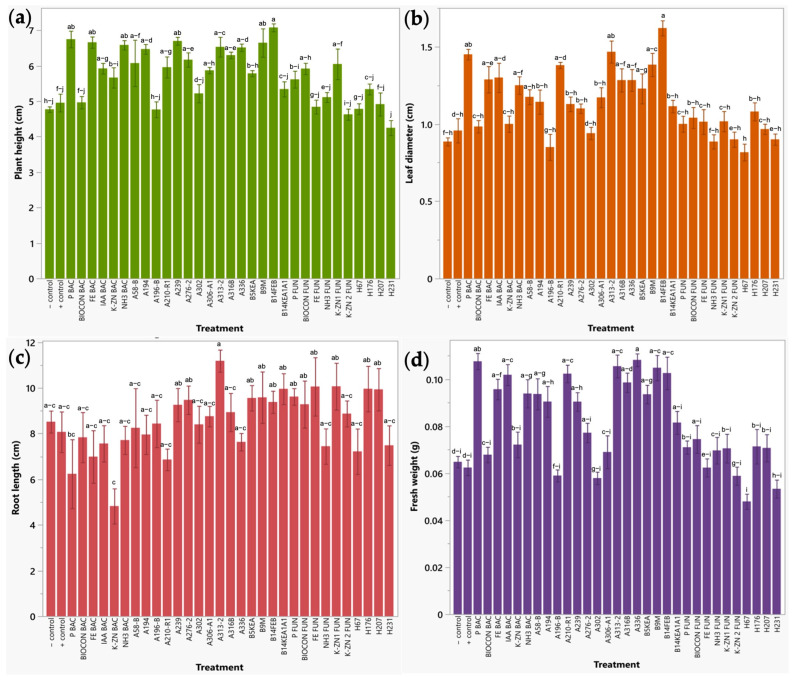
Evaluation of individual and combined application of microbial isolates on agronomic parameters in broccoli. (**a**) Plant height (cm). (**b**) Leaf diameter (cm). (**c**) Root length (cm). (**d**) Fresh weight (g). Results are expressed as mean ± standard deviation, letters indicate significant difference between treatments (*p* < 0.05).

Plant height was significantly improved by microbial consortia (P BAC, FE BAC, NH3 BAC, IAA BAC, K-ZN1 FUN, and BIOCON FUN) and individual bacterial isolates (B14FEB, A239, B9M, A313-2, A336, A194, A316B, A276-2, A58-B, A210-R1, and A306-A1) between 23.06 and 48.43% as compared with untreated plants. Of these, bacterial treatments B14FEB, P BAC, A239, FE BAC, B9M, NH3 BAC, A313-2, A336, A194, A316B, and A276-2 also presented a significant increase from 24.49 to 42.82% with respect to chemical fertilized plants (positive control). Leaf diameter exhibited significant increases between 32.66 and 82.91% when treated with bacterial bioformulations (consortia (P BAC, IAA BAC, FE BAC, and NH3 BAC) and individual isolates (B14FEB, A313-2, B9M, A210-R1, A316B, A336, B5KEA, and A58-B)) compared to water-irrigated plants (negative control). Particularly, the treatments B14FEB, A313-2, P BAC, and B9M increased leaf diameter up to 69.26% compared to positive control. Root length did not lead to significant results. This parameter presented high variability in the growth of secondary and main roots, probably caused by the size of the seedbed cavities. The roots were tangled and were managed carefully to avoid root breakage and loss of plant material that could affect weighing. Finally, fresh weight was also significantly increased by bacterial bioformulations, including A336, P BAC, A313-2, B9M, B14FEB, A210-R1 and IAA BAC as compared to both negative (57.27–67.03%) and positive (63.32–73.45%) controls (Figure 3). No significant difference was observed between the negative (water-irrigated plants) and positive (chemical-fertilized plants) controls in any agronomic parameter; this may be due to the initial nutrient load in the substrate. As the manufacturer indicates, black soil is a substrate rich in nutrients, soil minerals, and organic matter, which contribute to the overall plant health and growth. 

#### 2.2.2. Early Plant Response of Radish Sprouts to Bio-Formulations in a Seedbed Assay

**Figure 4:**
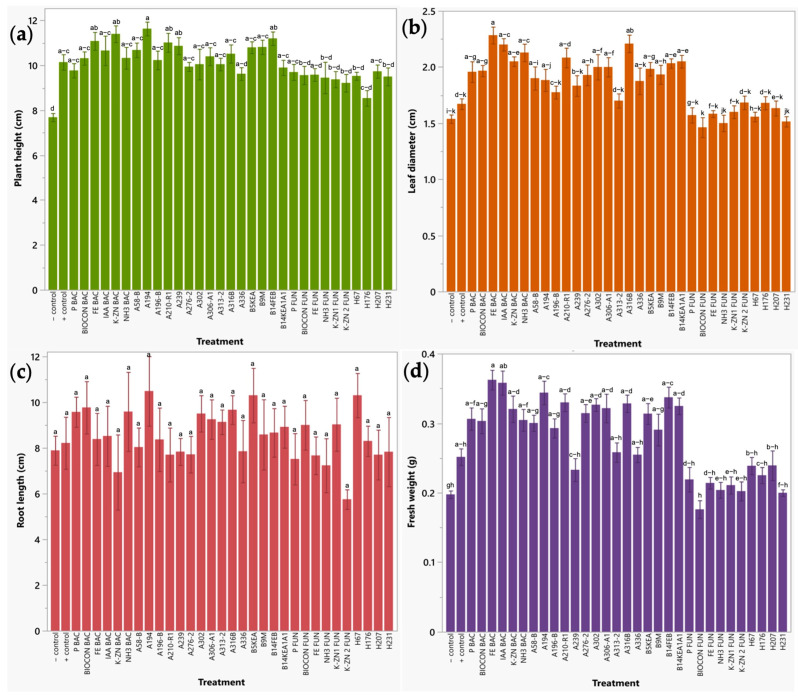
Evaluation of individual and combined application of microbial isolates on agronomic parameters in radish. (**a**) Plant height (cm). (**b**) Leaf diameter (cm). (**c**) Root length (cm). (**d**) Fresh weight (g). Results are expressed as mean ± standard deviation, letters indicate significant difference between treatments (*p* < 0.05).

Plant height of radish was mainly influenced by bacterial treatments (consortia (K-ZN BAC, FE BAC, IAA BAC, NH3 BAC, BIOCON BAC, and P BAC) and individual isolates (A194, B14FEB, A210-R1, A239, B9M, B5KEA, A58-B, A316B, A306-A1, A196-B, A313-2, A302, A276-2, and B14KEA1A1). They enhanced plant growth by 26.18 to 51.32%; only fungal isolate H207 and fungal consortia P FUN increased this parameter compared to untreated plants. The same bacterial bioformulations significantly improved leaf diameter; it increased between 25.43 and 48.51% and 27.35 and 36.61% compared to negative and positive control, respectively. As in broccoli, root length measurements did not yield conclusive results. Finally, five bacterial consortia (FE BAC, IAA BAC, K-ZN BAC, P BAC, and NH3 BAC) and nine isolates (A194, B14FEB, A210-R1, A316B, A302, B14KEA1A1, A306-A1, A276-2, and B5KEA) also enhanced plant fresh weight between 54.18 and 85.45% as compared to water-irrigated plants (Figure 4).

### 2.3. Identification of Potential PGP Microbial Isolates

Based on previous in vivo assays where bacteria were the most efficient in improving plant growth, the best nineteen PGP bacterial isolates were identified through DNA sequencing (Table 5). Identified isolates corresponded mostly to species belonging to the genera *Pseudomonas* (seven isolates) and *Pantoea* (four isolates) from soil and root samples of pepper, maize, greasewood, and oregano. It is important to notice that samples corresponding to the same identified species presented similar levels of plant growth-promoting traits in Table 3, as in the case of *Serratia liquefaciens* strain A302 and B14FEB, and *Pseudomonas arenae* strain A211 and A196.

**Figure 5:**
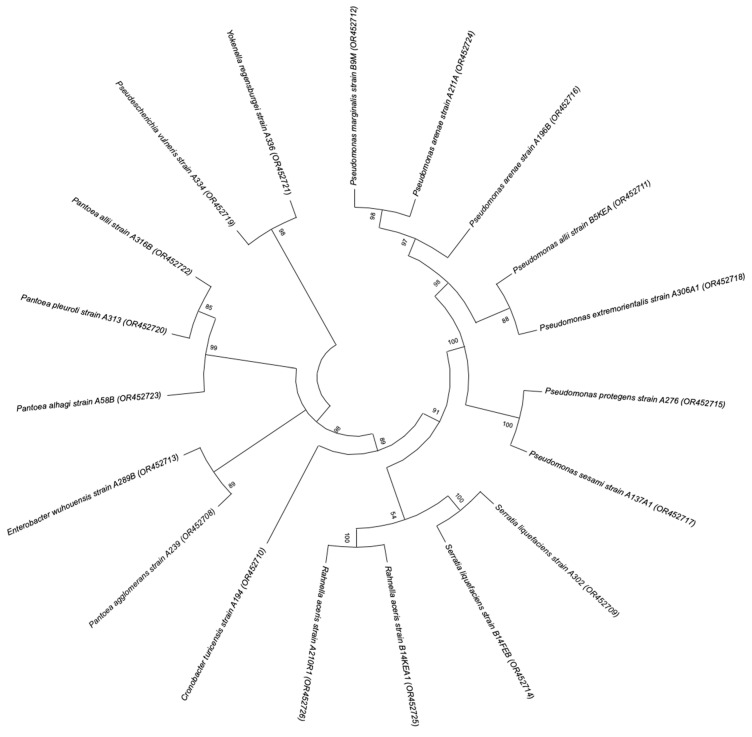
Neighbor-joining phylogenetic tree constructed based on the 16S rDNA sequences showing relationships between the bacterial isolates. Numbers at nodes denote the bootstrap support levels based on data for 1000 replicates. Accession numbers are given in parenthesis.

A phylogenetic tree was constructed based on the 16S rDNA gene sequences of the bacterial isolates. As observed in Figure 5, most of the species belong to the genus *Pseudomonas*, which were grouped in the same clade, being closer to *Serratia* and *Rahnella* species than to the rest of the isolates. *Pantoea* and *Enterobacter* species were also grouped in the same clade, which along with *Pseudescherichia*, *Yokenella*, and *Cronobacter* species belong to the Enterobacteriaceae family.

## 3. Discussion

To develop effective biofertilizers that benefit agriculture, environment, and farmers’ economies, an initial comprehensive laboratory study is required to screen microbial species with important plant growth-promoting traits [1]. The selection of potential PGPR strains is a big challenge. First, it is important to consider the source of the microbial strains that will be tested since exposure to different plant root exudates, tillage practices, fertilization protocols, and climatic conditions exert selective effects on soil microbial communities [10]. Therefore, in this study, different croplands and wild plant species were sampled to increase the diversity of the isolated microorganisms. In addition, an arid climate was chosen since plants growing in arid and semiarid regions are exposed to harsh environmental stresses, including soil nutrient scarcity, water deficiency, high temperature, and solar radiation, and can survive, to a large extent, due to their association with beneficial microorganisms which are genetically and metabolically adapted to grow in these conditions and can help host plants to maintain homeostatic balance [7,12,13].

In the present work, the findings and recommendations of different authors on the methodology for the isolation and selection of PGPM were considered [4,6,14], particularly those of Vasseur-Coronado et al. [6] described previously. In the step of isolation of microbial candidates, it was decided to follow the traditional approach of using a highly available commercial nutrient-rich growth media to recover culturable microbial strains, since even when it is known that a high number of microbial species are unable to proliferate on synthetic media because they require their natural growth environmental conditions, i.e., 99% of soil bacteria are unculturable [17], it is also important to consider that one of the key points for the production of PGPM inoculants is the viability and ease of mass multiplication to ensure that a sufficient number of viable cells can colonize the plants’ rhizosphere [18]. 

Herein, numerous microbial species were isolated and evaluated for multiple plant growth-promoting attributes. At the species level, plant growth promotion mechanisms differ among beneficial microorganisms [1]. Thus, the characterization of the mode of action of isolated PGPM is an important phase when selecting microbial species for biofertilization purposes [6]. After several rounds of selection, it was found that microorganisms had greater mineral (P, K, Zn) solubilizing activity than other growth-promoting parameters such as phytohormone production, nitrogen fixation, ammonia secretion, production of Fe-chelating compounds, and biocontrol activity. This result may be because phosphorus and potassium are crucial macronutrients for both bacteria and plants; both organisms acquire these minerals from inorganic sources in the surrounding soil environment [19]. 

Phosphorus and potassium usually form insoluble mineral compounds with calcium, iron, aluminum, or manganese. These insoluble nutrient forms are unavailable for microorganisms and plants and must be solubilized to increase their bioavailability. The main mechanism implicated in their solubilization involves the synthesis and secretion of organic acids by soil microorganisms. These organic acids are commonly produced from glucose oxidation and are involved in primary metabolism and normal microbial growth and development [11,20,21]. Other microbial mechanisms of plant growth promotion may be triggered by different factors, including the presence of specific nutrients in the root exudates such as amino acids, sugars, organic acids, and phenolic compounds, the recognition of signal molecules between the microorganisms and plant roots, the interaction with other microorganisms, and the exposure to stress-associated factors [3,12,14,20,21]. Optimization of the characterization protocols of the PGP attributes could also help to increase the expression of each trait. For example, Widawati [22] optimized the culture media for IAA quantification and found that *Bacillus siamensis* reached the maximum IAA production (9.89 μg/mL–16.61 μg/mL) at 96 h of incubation using a medium supplemented with tryptophan (250 ppm), sucrose, and tryptone. 

In general, the obtained results were similar to other studies, Marwanto et al. [23] evaluated several plant growth-promoting traits in seven bacterial isolates and five fungal isolates (*Paecilomyces* sp., *Aspergillus niger*, *Trichoderma* sp., *Saccharomyces* sp., and *Aspergillus nidulans*). They also found that isolates presented mineral-solubilizing activity, but no nitrogen fixation activity was observed in fungal isolates using the NFB medium. Abedinzadeh et al. [24] identified that P-solubilizing fungal isolates have larger halo solubilization zones (1.0–14.0 mm) than bacteria (0.5–6 mm), as observed by Marwanto et al. [23] and the present study. Mpanga [25] found IAA production levels of 74–132 mg/L for rhizosphere bacteria. In this work, bacterial IAA was quantified in ranges between 29 and 176 mg/L. ALKahtani et al. [7] also isolated and characterized bacterial endophytes with phosphate-solubilizing clearing zones ranging from 7.6 ± 0.3 to 9.6 ± 0.3 mm. Additionally, isolates secreted 60 mg/L of IAA in broth media supplemented with tryptophan (5 mg/mL). Bhattacharyya et al. [26] tested NH_3_ production of 30 rhizobacteria isolated from tea plants, NH_3_ was quantified (2.5 μmol mL^−1^ to 7.54 μmol mL^−1^) in all the isolates. Isolates from nodules of *Mimosa pudica* have shown positive results in siderophore production, mainly *Bacillus cereus* CUAMS116, along with *Enterobacter* species and *Serratia* sp. [27].

Basu et al. [1] reported different criteria that a soil microorganism should fulfill to be considered an ideal PGPM. They include adequate adaptation and compatibility with the rhizosphere and the microbial communities involved, high plant root colonization rates upon inoculation, significant capability to promote plant growth, a wide spectrum of action avoiding selective or highly targeted strains to specific crops, great tolerance to abiotic conditions such as drying, oxidants, heat, and radiation, and increased competitive skills over existing microbial communities. Therefore, the following evaluation steps involved the microbial mass propagation on a commercial growth medium and further in vivo bioassay on two different plants species grown under seedbed conditions to identify plant pathogenicity or the establishment of symbiotic rhizosphere colonization capable of promoting plant growth and development, since successful plant growth promotion implies that PGPM colonized and interacted adequately with plant roots in the rhizosphere [28]. Since combined PGP traits were considered in the selection of the best strains for biofertilizer formulation, it was decided to evaluate fungi mostly as consortia since some fungal species, including species of the genera *Aspergillus* and *Penicillium*, are considered facultative microorganisms or latent plant pathogens associated with various plant diseases such as anthracnose, plant rot, leaf spot, wilt, blight, mildew, and post-harvest diseases, among others [29,30,31,32]. Bacterial strains individually exhibited a better combination of plant growth-promoting mechanisms, making them potential candidates for the formulation of biofertilizers. Therefore, their individual evaluation was relevant for discriminating between the isolates and identifying possible negative interactions when they were applied as consortia.

Regarding in vivo studies, bacteria had significantly (ANOVA, *p* < 0.05) better effects on plants than fungi. All bacterial consortia notably improved all agronomic parameters except for BIOCON BAC. Individual application also generated significant increases, but as previously mentioned, it is advisable to apply them as consortia to take advantage of their complementary effect and ensure their survival when applied in the field [9,11,33]. Other reports have also revealed significant improvements in the vegetative growth of broccoli and radish when using biofertilizers, similar to those obtained in these experiments [34,35,36]. Agustiyani et al. [34] isolated fifteen bacterial isolates and performed in vitro screening for several PGP activities. They found that isolates were positive for IAA production (10.2–125.3 mg/L) (seven isolates), phosphate solubilization (ten isolates), nitrogen fixation (ten isolates), ACC (1-aminocyclopropane-1-carboxylate)-deaminase enzymatic activity (12 isolates), siderophore secretion (seven isolates), and ammonia production (nine isolates). PGPM were evaluated in radish grown under greenhouse conditions, and all the bacterial strains significantly increased the growth and tuber formation compared to control. Fresh shoot weight was increased by up to 206.75%.

Some fungal treatments even negatively impacted plant growth in almost all parameters, as in the case of treatments H231, K-ZN 2 FUN, and H67. As fungi are macrostructures compared to bacteria, there can be an interspecific competition between plants and microorganisms for the same nutrient forms; soil microorganisms can outcompete plants for more soil nutrients [19,20]. However, fungi showed important biocontrol activity. Thus, further research is needed to evaluate this effect in plants and the combined application of these isolates with bacteria to exploit both mechanisms: bacterial plant growth promotion and fungal biocontrol activity. For example, Abro et al. [37] also evaluated endophytic fungal species against *Fusarium oxysporum* f. sp. *cucumerinum;* results revealed that 30 fungal isolates inhibited the mycelial colony growth of *Fusarium* over 66%, especially *Hypocrea* sp. and *Penicillium* sp., were highly effective against this phytopathogen; greenhouse studies also exhibited that the antagonistic fungal isolates successfully inhibited the wilt disease severity. 

The last characterization step of this work consisted of the taxonomic identification of the best PGP isolates. Considering that the rhizosphere involves a small number of beneficial microorganisms, for example, only a few species of *Bacillus* spp. out of hundreds within the genus show multiple PGP traits and might be useful for biofertilizer formulations. Identifying microbial species is very relevant because the beneficial activity is characteristic of certain species [14]. Bacterial species found in this work belonging to the genera *Enterobacter*, *Pantoea*, *Pseudomonas*, and *Serratia* have been successfully studied as PGPM in several crops [4,5,8,38,39,40,41,42,43,44,45,46,47,48,49]. In the case of *Pseudomonas allii*, it has also been reported as a plant pathogen responsible for soft rot in onions [50]; even though the plants in this study did not show disease symptoms, further experiments must be performed at later stages of plant development to determine their safety. *Rahnella aceris* was recently described as a novel PGP rhizobacterium [51], as well as *Cronobacter turicensis* and *Yokenella regensburgei*, which have not been widely studied for biofertilization purposes; this work contributes to the understanding of their role in improving plant growth and development [52,53,54]. 

Proper identification and characterization of microorganisms is also important when formulating microbial consortia [4,6,13]. The microorganisms in an inoculum can develop several types of interactions (e.g., synergy or competition) depending on factors such as microbial diversity, environmental conditions, and culture medium [3,11,13]. There are several cases in which microbial identification would help to understand the dynamics of these interactions, for example, when (1) certain combinations of microorganisms promote the dominance of certain species and suppress the growth of others, (2) members with greater taxonomic proximity share resource requirements favoring their cultivation, (3) different microbial genera develop complementary cross-feeding functions in which the secreted metabolites of one species function as a substrate for others, (4) the release of signaling molecules from certain species help shape population density and behavior, or when (5) certain microorganisms alter environmental conditions (pH, oxygen content, viscosity) that influence the growth and development of the consortium [2,3,4,5,11,13]. Thus, the complete taxonomic study of the consortia can significantly help to enhance overall performance and function of biofertilizers. Further tests of the interactions of the species with the greatest plant growth-promoting potential are required to optimize the microbial consortia evaluated in this study.

## 4. Materials and Methods

### 4.1. Sampling

**Figure 6:**
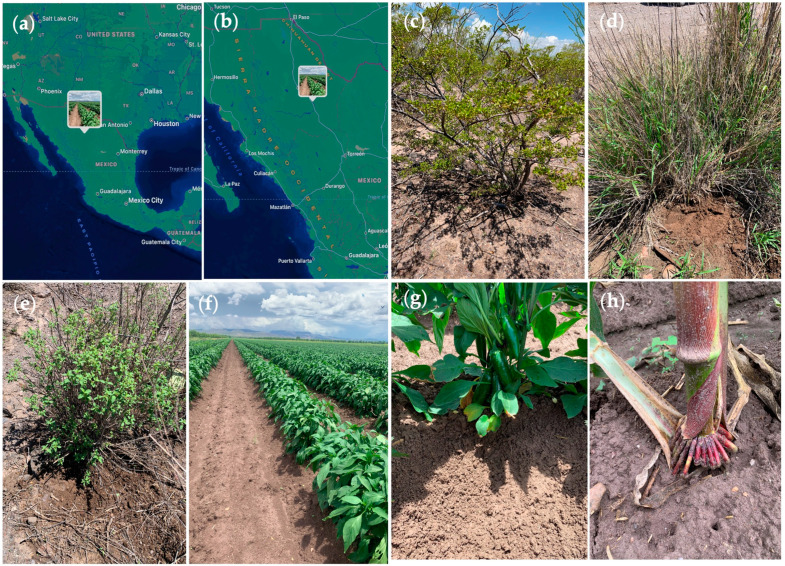
Root sampling in agri-food crops (maize and pepper) and wild plant species (oregano, greasewood, and buffelgrass). (**a**,**b**) Sampling sites in south-central Chihuahua. (**c**) Greasewood sampling (20 cm depth). (**d**) Buffelgrass sampling (20 cm depth). (**e**) Oregano plant sampled at 20 cm depth. (**f**) Pepper sampling site located in Saucillo town (south-central Chihuahua). (**g**) Pepper sampling (10–15 cm depth). (**h**) Maize sampling (12 cm depth).

Two agri-food crops (maize (*Zea mays*) and jalapeño pepper (*Capsicum annuum*)) and three wild plant species (oregano (*Origanum vulgare*), greasewood (*Larrea tridentata*), and buffelgrass (*Cenchrus ciliaris*)) were sampled at two different fields each located in a south center region (28.08 N 105.33 W) of the State of Chihuahua, Mexico (Figure 6) in July 2021. All the ten sampled fields were distributed in the same town (Saucillo) and were located around 5 to 15 km from each other. Plant species were chosen depending on the region’s crop availability, abundance, and economic relevance. This region is situated within the Chihuahuan desert and is characterized by strong agricultural activity; it has an arid climate with a mean annual temperature ranging from 12 °C to 31 °C, while the mean annual precipitation is 292 mm with a range of about 150–500 mm [55,56,57]. 

Sampled croplands were exposed to traditional agricultural practices. They were crop rotated with watermelon, maize, and alfalfa and chemically fertilized with urea (NPK 46-00-00), diammonium phosphate (NPK 18-46-00), and potassium chloride (NPK 0-0-60), applying three to four times per crop cycle depending on the plant developmental stage and the farmer. Irrigation was made every 10–15 days via surface irrigation.

In total, 30 samples were collected and processed, corresponding to roots and rhizospheric soil of five plant species from two fields with three replicates. Each sample consisted of approximately 250 g of soil and roots and was taken with a shovel at 20 cm depth for greasewood, buffelgrass, and oregano, and 10–15 cm depth for pepper and maize. The shovel was cleaned between samples to avoid cross-contamination. Samples were stored in sealed plastic bags at 4 °C until processing.

### 4.2. Isolation of Native Soil Microorganisms

Samples were homogenized with a vortex mixer for 30 s, then 1.7 g of homogenized soil of each replicate from each field was mixed to obtain a 5 g composite sample. It was mixed with 45 mL of sterile distilled water and serially diluted with ten-fold dilutions (10^−2^–10^−3^). Three replicates per field were prepared. From each dilution, 100 µL were inoculated on solid enrichment growth media (tryptic soy agar (TSA) for bacteria, and potato dextrose agar (PDA) for fungi). To recover endophyte microorganisms from roots samples, roots were washed with distilled water to remove soil particles. Then, they were surface sterilized with 70% ethanol for 5 min, followed by 1% sodium hypochlorite for 10 min, and washed three times with sterile distilled water. To verify the effectiveness of the disinfection method, 50 µL of the water from the last wash was inoculated in TSA and PDA growth media. For each sample, eighteen fragments of approximately 1 cm from the sterilized roots were cut, and three fragments were placed in the same solid culture media for fungi and bacteria [58,59]. Colonies were selected based on visual morphological differences, e.g., pigmentation, opacity, form, elevation, texture, and margin. They were subcultured by transferring into the same agar medium to obtain pure colonies. Not all colonies growing on agar plates were isolated and purified [60]. 

### 4.3. Evaluation of Plant Growth-Promoting Traits

Isolated bacterial and fungal species were evaluated by qualitative and quantitative assays for several plant growth-promoting traits, including biocontrol activity. The PGP properties that were evaluated are potassium, phosphate, and zinc solubilization, nitrogen fixation, ammonia production, indole-3-acetic acid (IAA) secretion, siderophore production, and antifungal activity against phytopathogenic *Fusarium oxysporum* [61]. 

#### 4.3.1. Potassium, Phosphate, and Zinc Solubilization

Isolates were directly inoculated in three differential culture media to identify microorganisms with the desired metabolic characteristics and ability to promote plant growth. Potassium solubilization was assessed using Aleksandrov agar medium (glucose: 5 g L^−1^, MgSO_4_.7H_2_O: 0.5 g L^−1^, FeCl_3_: 0.005 g L^−1^, CaCO_3_: 0.1 g L^−1^, Ca_3_(PO_4_)_2_: 2 g L^−1^, AlKO_6_Si_2_: 2 g L^−1^, Agar: 20 g L^−1^, pH 7) [62,63]. Phosphate solubilization activity was evaluated using Pikovskaya agar medium (dextrose: 10 g L^−1^, yeast extract: 0.5 g L^−1^, Ca_3_(PO_4_)_2_: 5 g L^−1^, KCl: 0.2 g L^−1^, MgSO_4_.7H_2_O: 0.1 g L^−1^, MnSO_4_.7H_2_O: 0.0001 g L^−1^, FeSO_4_.7H_2_O: 0.0001 g L^−1^, (NH_4_)_2_SO_4_: 0.5 g L^−1^, Agar: 15 g L^−1^, pH 7) [64,65,66,67]. Similarly, zinc solubilization was measured using modified PVK agar medium (glucose: 10.0 g L^−1^, (NH_4_)_2_SO_4_: 1 g L^−1^, KCl: 0.2 g L^−1^, K_2_HPO_4_: 0.2 g L^−1^, MgSO_4_.7H_2_O: 0.1 g L^−1^, yeast extract: 0.2 g L^−1^, ZnO: 1 g L^−1^, Agar: 15 g L^−1^, pH 7) containing 0.1% insoluble zinc oxide [60,68]. Ten isolates were inoculated per plate using an inoculation loop to evaluate bacteria. For fungi, culture media were supplemented with chloramphenicol (0.1 g L^−1^) to inhibit bacterial growth; four isolates were inoculated on each plate, placing agar disks (5 mm in diameter) of 10-day old sporulated fungal cultures in PDA medium using a sterile scalpel. Plates were incubated 5 days at 30 °C for bacteria, and 10 days at 25 °C in the dark for fungi. 

Potassium-, phosphate-, and zinc-solubilizing microorganisms were identified by the presence of a clear halo zone around the colonies [59,62,66,67,69,70,71,72]. Colonies with visual morphological differences and positive activity to the growth-promoting traits were selected and subcultured into the same medium for the complete isolation of microorganisms and evaluation of the activity level by halo size measurements. Isolates were classified by the size of the halo. Halos were measured after 4 days for phosphorus and zinc, and 6 days for potassium solubilization; they were sized from the margin of the colonies and classified into three levels: level 1 (1 mm), level 2 (2–4 mm), and level 3 (≥5 mm). Colonies with greater solubilizing activity (level 3) were subcultured in an enrichment medium (TSA and PDA) to ensure pure colonies. Bacterial isolates were Gram-stained to study microbial morphology and discard possible contamination. Final isolates were stored at 4 °C for further analysis.

PGP screening tests were repeated two months later to ensure consistency and stability in the levels of the plant growth-promoting mechanisms of selected isolates. In case of important changes or contamination, isolates were subcultured in an enrichment medium (TSA for bacteria and PDA for fungi) and reevaluated until activity corresponded to the first results.

#### 4.3.2. Nitrogen Fixation

Microbial growth evaluation in nitrogen free medium was used as a starting point to identify nitrogen-fixing microorganisms, as it helps to differentiate microbial strains capable of fixing atmospheric nitrogen from those that depend on external nitrogen sources. Further tests are required to confirm nitrogen fixation activity of the isolates; these assays can involve the evaluation of the nitrogenase enzymatic activity or the expression of nitrogen-fixing genes [13,59]. Isolates were inoculated in N-free solid malate medium (Nfb) (malic acid: 5 g L^−1^, KOH: 4 g L^−1^, K_2_HPO_4_: 0.5 g L^−1^, FeSO_4_.7H_2_O: 0.05 g L^−1^, MnSO_4_.7H_2_O: 0.01 g L^−1^, MgSO_4_.7H_2_O: 0.01 g L^−1^, NaCl: 0.02 g L^−1^, CaCl_2_: 0.01 g L^−1^, Na_2_MoO_4_: 0.002 g L^−1^, Agar: 15 g L^−1^, pH 6.8, 5 mL 1% Bromothymol blue (BTB) solution). Nitrogen-fixing microorganisms were identified by a color change in the medium from pale green to blue as a result of the increase in the pH of the medium due to the microbial conversion of N_2_ into ammonia (NH_3_), BTB solution was used as a pH indicator to allow visualization of the color halos [59,70,71]. Halos were measured after 4 days and were classified as level 1 (<10 mm), level 2 (10–14 mm), or level 3 (≥15 mm). Colonies with greater activity levels were identified and transferred to an enrichment medium (TSA and PDA) to ensure pure colonies. Bacterial isolates were stored at 4 °C until use. 

#### 4.3.3. Ammonia Production

For bacterial evaluation, a single colony of the final isolates was grown in 5 mL of trypticase soy broth (TSB) and incubated on a rotary shaker at 180 rpm and 30 °C for 48 h. Optical density was measured at 600 nm (OD_600_) with a microplate reader VARIOSKAN LUX (ThermoFisher Scientific, Waltham, MA, USA) to estimate the colony forming units (CFU) per mL using a conversion equivalence of 0.5 OD_600_ corresponding to 1 × 10^8^ CFU/mL. From each sample, 1 × 10^6^ CFU/mL was inoculated in 1 mL of peptone water at 30 °C and 180 rpm for 72 h. In the case of fungi, 10-day-old fungal agar disks of 5 mm in diameter were inoculated in 10 mL of peptone water at 25 °C in darkness for 7 days. Cultures were centrifuged at 10,000 rpm for 10 min, and 1 mL of the supernatant was added with 50 µL Nessler reagent (HgCl_2_: 100 g L^−1^, KI: 70 g L^−1^, NaOH: 160 g L^−1^, pH 13). The reaction tube was incubated for 30 min in the dark at room temperature for color change. The development of brown-orange color was quantified by plate reader at 425 nm (OD_425_). Uninoculated peptone water was used as blank [60,73,74,75]. 

#### 4.3.4. Indole-3-Acetic Acid Secretion

A volume of 20 µL at 1 × 10^6^ CFU/mL of each sample was inoculated in 1 mL of sterile trypticase soy broth (TSB) supplemented with sterile L-tryptophan (800 µg/mL). L-tryptophan was dissolved in distilled water and sterilized using a 0.22 µm membrane filter. Bacterial cultures were incubated in the dark at 180 rpm and 30 °C for 72 h and then centrifuged at 10,000 rpm for 10 min. An amount of 150 µL of supernatant were transferred to a 96-well plate and mixed with 150 µL of Salkowski reagent (2 mL 0.5 M FeCl_3_, 49 mL distilled water, and 49 mL 70% perchloric acid (HClO_4_)). The microplate was incubated in the dark at 25 °C for 30 min, and optical density was measured at 530 nm (OD_530_). The concentration of IAA was estimated by a standard IAA curve. Each isolate was tested in triplicate [59,64,69,70,71,73,76,77,78,79]. 

Likewise, one fungal disk (5 mm in diameter) of 10-day-old sporulated cultures in PDA medium was inoculated into 5 mL of sterilized potato dextrose broth (PDB) supplemented with sterile L-tryptophan (2 mg/mL) and incubated in the dark in an orbital shaker at 130 rpm and 20 °C for 7 days. The supernatant was recovered by centrifugation at 10,000 rpm for 10 min. Then, 150 µL of the supernatant was mixed with 150 µL of Salkowski reagent and incubated in the dark at 25 °C for 30 min. Absorbance at 530 nm was measured to detect pink coloration. Three replicates were made [80]. 

Colorimetric reaction of IAA with salkowski reagent was used as a quick and simple preliminary screening assay for IAA production. It is important that for accurate quantitative evaluation of IAA production, this method should be complemented with analytical techniques such as high-performance liquid chromatography (HPLC) or gas chromatography–mass spectrometry (GC-MS), since salkowski reagent can react with other compounds containing indole rings [59,76,79,80]. 

#### 4.3.5. Siderophores Production

The siderophore activity of microbial isolates was determined qualitatively by the chrome azural sulfonate (CAS) agar plate method, as described by Srimathi and Suji [81]. CAS medium was supplemented with hexadecyltrimethylammonium bromide (HDTMA), a cationic solvent that stabilizes the Fe–CAS complex. Ten bacterial strains were inoculated per plate with an inoculation loop and incubated for four days at 30 °C. For fungi, four isolates (fungal agar disks of 5 mm in diameter) were placed on the CAS agar and incubated for 10 days in the darkness at room temperature. The development of an orange-yellowish clearing zone will indicate siderophore production. Isolates were classified based on halo size into three levels: level 1 (1 mm), level 2 (2–4 mm), and level 3 (≥5 mm) [60,64]. 

#### 4.3.6. Antifungal Activity against Phytopathogenic *Fusarium oxysporum*

The dual culture assay was used to measure the antimicrobial activity of the isolated strains. Previously isolated and characterized fungal plant pathogen *Fusarium oxysporum* was grown on PDA plates at 25 °C for 10 days. Fungal disks were obtained using the back side of sterile 200 μL micropipette tips. To test bacteria, disks were placed in the center of new PDA plates, and the selected isolates were streaked 2 cm apart from the pathogenic fungus. Plates were incubated at 28 °C and checked for the formation of a zone of clearance for 7 days. The size of the clearing zone indicated the potential for biocontrol activity. Plates inoculated with the pathogen but without microbial isolates were used as the positive control [60,73].

In the case of fungi, disks of both phytopathogen and isolated fungi were placed in opposite sites near the plate’s margins and incubated at room temperature in the dark for 7 days. Qualitative results were registered for bacterial antifungal activity, and fungal growth inhibition of fungal isolates was assessed by calculating the inhibition percentage in radial growth (% IRG) [82].

### 4.4. Preparation of Bacterial and Fungal Bio-Formulations 

Bacterial and fungal isolates that showed good performance for multiple plant growth promoting traits (K, P and Zn solubilization, nitrogen fixation, NH_3_ production, siderophore biosynthesis, IAA production, and biocontrol activity) were selected and propagated to prepare bioformulations. For bacteria, a total of 24 isolates were individually grown in TSB enrichment medium and incubated on a rotary shaker at 180 rpm and 30 °C for 48 h [59,83,84]. Bacterial biomass production was monitored spectroscopically until reaching optical density (600 nm) between 0.6 and 0.8, which corresponded to the total plate counts of 1.2 × 10^8^–1.6 × 10^8^ CFU mL^−1^ [59,83,85]. Bacterial growth was also confirmed by plate count in TSA agar plates. Serial dilutions (10^0^–10^−4^) of the cultures were made in sterile distilled water; 20 µL of each dilution were inoculated on TSA agar plates and incubated for 24 h at 30 °C. The dilution in which bacteria formed between 30 and 300 colonies was considered. To estimate cell density, the number of colonies was multiplied by the correction factor to adjust the count to 1 mL (50, in this case) and by the inverse of the dilution used [86,87,88,89,90].

Likewise, fungal inocula were prepared by growing the best isolates exhibiting several PGP traits. Inoculants were based on spores of 10-day-old PDA fungal cultures. Each culture plate was added with 5 mL of sterile distilled water. The spore suspension was collected by scratching the fungus surface on the Petri dishes. Eighteen isolates were considered, transferred to sterile 50 mL falcon tubes, and adjusted to the maximum volume. A Neubauer chamber was used to perform the spore count [65,91,92]. Bioformulations were stored at 4 °C until use. 

### 4.5. In Vivo Evaluation of Bio-Formulations in a Seedbed Assay

A seedbed experiment was performed in a growth chamber for 12 days to evaluate the early plant response to microbial isolates in radish (*Raphanus sativus*) and broccoli (*Brassica oleracea*). The experiment was conducted using a completely randomized design. Nineteen microbial isolates (fifteen bacteria and four fungi) and twelve consortia (six bacteria and six fungal consortia) were tested. Bacterial and fungal consortia were prepared by classifying selected isolates according to the growth promoting traits, and treatments were the following: (1) P-solubilizing bacteria. (2) P-solubilizing fungi. (3) K- and Zn-solubilizing bacteria. (4) K- and Zn-solubilizing fungi. (5) K- and Zn-solubilizing fungi 2. (6) N-fixing and NH3-producing bacteria. (7) N-fixing and NH3-producing fungi. (8) Siderophore-producing bacteria. (9) Siderophore-producing fungi. (10) Biocontrol bacteria. (11) Biocontrol fungi. (12) IAA secreting bacteria. Consortia contained the best microbial isolates for the PGP traits of each treatment; equal volumes of each microbial suspension were mixed according to the treatment formulation. Controls were uninoculated plants irrigated with water (−control) and uninoculated plants irrigated with water and chemical fertilizer solution (Hoagland solution) (+control). Three replicates (cavities) were considered per treatment, and nine plants per treatment were evaluated. Experiments were carried out in plastic seedbeds of 72 cavities (5 cm in diameter and 6 cm deep), filled to ¾ of their capacity with sterile black soil [93,94]. 

Inoculants were evaluated in radish (*Raphanus sativus* L. var. ‘Champion’) and broccoli (*Brassica oleracea* L. var. *italica*), using commercial seeds (Vita^®^) from “Rancho Los Molinos” company. Seeds were surface sterilized by soaking them in 70% ethanol for 30 s, followed by 5% sodium hypochlorite solution for 5 min, and five washes with sterile water. Seeds were soaked in sterile distilled water with aeration overnight in darkness at room temperature to induce germination [60,64]. Each cavity was sown with ten seeds at 0.5–1 cm depth [59]. Plants were watered daily with tap water [93,95].

Biofertilization treatments were applied three times during the experiment: days 1, 6, and 9, applying two mL of the biofertilizer to the corresponding cavities of each treatment in the seedbed. Final concentrations of 10^8^ CFU mL^−1^ and 10^6^ spores mL^−1^ were used for bacteria and fungi, respectively. Plants were harvested on day 12, and agronomic attributes were measured [60,64,84,92,96,97]. 

Agronomic growth parameters of plant height (cm), root length (cm), leaf diameter (cm), and shoot fresh weight were determined [61,64,98]. Length was recorded from the base to the tip of the plants [99]. Collected measurements were averaged to express the results. Data was subjected to statistical analysis by one-way analysis of variance (ANOVA) followed by Tukey Test at *p* < 0.05 to compare mean values. *Jmp* software (version 17.0) was used. Results were expressed as mean ± standard deviation (SD) [61,64,95,98]. 

### 4.6. Identification of Potential PGP Microbial Isolates

The best nineteen plant growth-promoting bacterial isolates were identified through DNA sequencing of the 16S rRNA gene. Bacteria were subcultured to obtain separated and pure colonies in standard enrichment solid medium without nutrient limitation; TSA was used for this purpose, and a single colony was transferred to 10 mL of TSB and incubated at 180 rpm and 30 °C for 48 h. Genomic DNA was extracted using Promega Wizard^®^ Genomic DNA Purification kit (Promega, Madison, WI, USA), following manufacturer’s instructions. The 16S rRNA gene was amplified using the universal primers 27F (5′ AGAGTTTGATCMTGGCTCAG 3′) and 1492R (5′ GGTTACCTTGTTACGACTT 3′) reported by James [100]. PCR conditions were initial denaturation at 94 °C for 3 min, followed by 30 cycles at 96 °C for 15 s, annealing at 60 °C for 1.5 min, and extension at 72 °C for 2 min, with a final polymerization extension at 72 °C for 5 min. After PCR amplification, PCR products were verified by 1% agarose gel electrophoresis. Samples were subject to bidirectional DNA sequencing (Eurofins Genomics, LLC, Louisville, KY, USA). Raw 16S rDNA sequences were analyzed with the Basic Local Alignment Search Tool (BLAST) of the National Center for Biotechnology Information (NCBI). Sequence length of amplified 16S rRNA genes varied between 496 and 1029 nucleotides. Taxonomic assignment was performed, and taxa were clustered to build a phylogenetic tree using the Molecular Evolutionary Genetics Analysis (MEGA) software (version 11) [95]. 

## 5. Conclusions

The above experimental work validated a high-throughput approach for screening hundreds of rhizospheric microorganisms with plant growth-promoting potential isolated from roots and soil samples from agri-food crops and wild plant species. They were characterized regarding several PGP traits, showing great solubilization activity of insoluble mineral sources. After selecting candidates with desired features, its function to improve the growth of broccoli and radish sprouts was proved, being able to determine that bacterial isolates were more effective in increasing plant growth, while the fungal isolates were relevant as *Fusarium* biocontrol agents in vitro. Most species identified with biofertilizing potential belonged to the genera *Pseudomonas* and *Pantoea*. The discovery and evaluation of new native species of beneficial soil microorganisms are of great relevance for modern agriculture to restore the fertility and biological activity of the soil and reduce the consumption of chemical fertilizers by using microbial strains capable of improving plant nutrient use efficiency. Future steps of this research will involve the application of these potential biofertilizers under greenhouse conditions to evaluate their effects on crop productivity and the nutritional quality of food.

## Figures and Tables

**Table 1:** Screening of microbial isolates for multiple PGP traits.

Selection Level	Number of Isolates	% with Respect to Total Isolates	Criteria
**Bacteria**			
Total isolates	798	100%	Visual morphological differences.
1st selection	399	50%	P, K, and Zn solubilizing activity, even if it is minimal.
2nd selection	262	33%	Two parameters in level 3 (P, K, Zn) and nitrogen fixation activity.
3rd selection	137	17%	Multi-mechanisms (P, K, and Zn solubilization, N_2_-fixation, NH_3_, IAA, and siderophores production).
4th selection	43	5%	High activity levels among different parameters, evaluation of biocontrol activity.
5th selection	24	3%	Best plant growth-promoting traits
**Fungi**			
Total isolates	209	100%	Visual morphological differences.
1st selection	156	75%	P, K, and Zn solubilizing activity, even if it is minimal.
2nd selection	55	26%	P, K, and Zn solubilization, N_2_-fixation, NH_3_, IAA, and siderophores production.
3rd selection	34	16%	Second grouping depending on macroscopic morphological differences.
4th selection	18	9%	Best plant growth-promoting traits, including biocontrol activity.

**Table 2:** Criteria to classify plant growth-promoting mechanisms according to activity levels.

PGP Attribute	Level 1	Level 2	Level 3
Phosphate, potassium, and zinc solubilization (clearing halo)	1 mm	2–4 mm	≥5 mm
Siderophores production (color change halo)			
Nitrogen fixation (color change halo)	0–9 mm	10–14 mm	≥15 mm
Ammonia production (OD_425 nm_ values)	−0.2–1	1–1.6	≥1.7
Indole-3-acetic acid secretion (hormone concentrations in mg/L)	0–10 mg/L	11–25 mg/L	≥26 mg/L
Biocontrol activity (% inhibition in radial growth)	0–39%	40–59%	≥60%

**Table 3:** Summary of PGP-traits of the final selected isolates.

Isolate	Phosphate Solubilization (Halo in mm)	Potassium Solubilization (Halo in mm)	Zinc Solubilization (Halo in mm)	Siderophore Production (Halo in mm)	Nitrogen Fixation (Halo in mm)	Ammonia Production (OD_425nm_ Values)	Indole-3-Acetic Acid Production (mg/L)	Biocontrol (Pathogen Growth Inhibition)
**Bacteria**								
**A302**	5.0	10.0	9.0	3.0	12.0	1.0	34.4	0%
**A306-A1**	7.0	6.0	12.0	1.0	0.0	0.8	8.2	0%
**A334-1**	3.0	5.0	5.0	1.0	0.0	1.7	33.7	0%
**B14FEB**	8.0	9.0	7.0	5.0	14.0	1.3	12.3	57%
**B14KEA1**	3.0	9.0	5.0	6.0	1.0	1.4	41.2	12%
**B5-KEA**	3.0	13.0	8.0	1.0	17.0	1.2	29.2	10%
**B9M**	7.0	6.0	8.0	2.0	13.0	1.2	40.9	0%
**A276-2**	5.0	7.0	10.0	1.0	7.0	1.1	5.3	0%
**A137 A1**	6.0	5.0	5.0	1.0	13.0	1.2	7.4	0%
**A211 A**	2.0	4.0	5.0	3.0	14.0	1.9	24.0	15%
**A239**	1.0	8.0	9.0	2.0	12.0	1.9	24.1	13%
**A336**	6.0	6.0	8.0	0.5	6.0	2.1	39.1	0%
**A32-2**	3.0	5.0	0.0	5.0	19.0	0.7	17.1	0%
**A58 B**	0.0	9.0	0.0	19.0	14.0	1.1	15.9	0%
**A80**	1.0	9.0	0.0	9.0	17.0	0.8	18.0	13%
**A194**	0.0	11.0	0.0	7.0	5.0	2.3	10.7	18%
**A210 R1**	2.0	6.0	10.0	3.0	13.0	2.0	21.1	68%
**A328 B**	2.0	7.0	0.0	2.0	11.0	1.6	12.6	50%
**A316 B**	3.0	6.0	9.0	1.0	10.0	1.2	7.2	49%
**A96 B**	5.0	6.0	8.0	4.0	18.0	0.9	23.1	43%
**A313-2**	3.0	5.0	0.0	2.0	7.0	1.7	55.8	21%
**A289 B**	8.0	8.0	6.0	2.0	0.0	1.5	176.7	19%
**A196 B**	2.0	6.0	8.0	4.0	13.0	1.3	60.6	23%
**A107 A**	6.0	8.0	0.0	1.0	11.0	1.8	64.7	0%
**Fungi**								
**H97**	11.0	14.0	16.0	14.0	0.0	0.9	1.2	69%
**H74**	6.0	10.0	11.0	1.0	1.0	0.2	1.0	36%
**H38**	5.0	13.0	11.0	0.0	0.0	0.5	1.5	64%
**H132**	3.0	11.0	15.0	0.0	1.0	0.2	1.0	40%
**H49**	6.0	19.0	12.0	3.0	1.0	0.1	9.6	38%
**H176**	15.0	15.0	14.0	23.0	0.0	0.7	5.1	51%
**H133**	4.0	12.0	9.0	12.0	0.0	0.3	1.4	69%
**H153**	4.0	5.0	10.0	2.0	1.0	0.6	1.7	40%
**H231**	6.0	20.0	15.0	0.0	1.0	0.2	1.2	33%
**H176-D**	12.0	10.0	11.0	2.0	0.0	0.1	1.0	31%
**H163**	4.0	9.0	14.0	0.0	1.0	0.8	2.4	47%
**H207**	20.0	12.0	15.0	16.0	0.0	0.9	0.5	51%
**H204**	4.0	10.0	8.0	0.0	1.0	0.2	3.1	53%
**H81**	4.0	4.0	12.0	1.0	1.0	0.2	1.2	36%
**H202**	15.0	4.0	11.0	0.0	1.0	0.6	1.5	40%
**H67**	4.0	16.0	19.0	21.0	0.0	0.0	0.5	53%
**H56**	5.0	5.0	10.0	0.0	1.0	0.3	2.0	38%
**H56-Zn**	6.0	6.0	17.0	13.0	0.0	0.7	0.9	51%

Purple, orange, and green colors indicate levels 1, 2, and 3, respectively, for each PGP trait.

**Table 4:** Microbial consortia based on plant growth promoting traits of each isolate.

Consortia ID	Description	Microbial Consortia
P BAC	Phosphate-solubilizing bacteria	A302, A306-A1, A334-1, B14FE-B
K-ZN BAC	Potassium- and zinc-solubilizing bacteria	B14KEA1-A1, B5KE-A, B9M, A276-2
N-NH3 BAC	Nitrogen(N_2_)-fixing and ammonia(NH_3_)-producing bacteria	A137-A1, A211-A, A239, A336
FE BAC	Siderophore-producing bacteria	A32-2, A58-B, A80, A194
BIOCON BAC	Biocontrol bacteria	A210-R, A328-B, A316-B, A96-B
IAA BAC	Indole-3-acetic acid-secreting bacteria	A313-2, A289-B, A196-B, A107-A
P FUN	Phosphate-solubilizing fungi	H133, H97, H176
K-ZN FUN 1	Potassium- and zinc-solubilizing fungi 1	H204, H231, H132, H81, H74, H176-D
K-ZN FUN 2	Potassium- and zinc-solubilizing fungi 2	H67, H49
N-NH3 FUN	N_2_-fixing and NH_3_-producing fungi	H202, H153, H163, H56
FE FUN	Siderophore-producing fungi	H56-Z, H207
BIOCON FUN	Biocontrol fungi	H38

**Table 5:** Molecular identification of bacterial isolates.

No.	Isolate	Sample Description *	Molecular Identification	Accession Number
1	A302	Greasewood, soil, 2	*Serratia liquefaciens* strain A302	OR452709
2	A306-A1	Pepper, root, 3	*Pseudomonas extremorientalis* strain A306A1	OR452718
3	A334-1	Greasewood, soil, 1	*Pseudescherichia vulneris* strain A334	OR452719
4	B14FEB	Maize, soil, 2	*Serratia liquefaciens* strain B14FEB	OR452714
5	B14KEA1	Pepper, soil, 2	*Rahnella aceris* strain B14KEA1	OR452725
6	B5KEA	Pepper, soil, 1	*Pseudomonas allii* strain B5KEA	OR452711
7	B9M	Maize, root, 3	*Pseudomonas marginalis* strain B9M	OR452712
8	A276-2	Pepper, root, 3	*Pseudomonas protegens* strain A276	OR452715
9	A137-A1	Maize, soil, 2	*Pseudomonas sesami* strain A137A1	OR452717
10	A211-A	Maize, root, 1	*Pseudomonas arenae* strain A211A	OR452724
11	A239	Oregano, soil, 3	*Pantoea agglomerans* strain A239	OR452708
12	A336	Oregano, soil, 2	*Yokenella regensburgei* strain A336	OR452721
13	A58B	Greasewood, soil, 1	*Pantoea alhagi* strain A58B	OR452723
14	A194	Greasewood, root, 1	*Cronobacter turicensis* strain A194	OR452710
15	A210R1	Maize, root, 1	*Rahnella aceris* strain A210R1	OR452726
16	A316B	Pepper, soil, 2	*Pantoea allii* strain A316B	OR452722
17	A313-2	Oregano, root, 1	*Pantoea pleuroti* strain A313	OR452720
18	A289B	Buffelgrass, soil, 1	*Enterobacter wuhouensis* strain A289B	OR452713
19	A196-B	Maize root, 3	*Pseudomonas arenae* strain A196B	OR452716

* Samples are described with plant source, sample type, and field number.

## Data Availability

Not applicable.
